# Norms of Interocular Circumpapillary Retinal Nerve Fiber Layer Thickness Differences at 768 Retinal Locations

**DOI:** 10.1167/tvst.9.9.23

**Published:** 2020-08-12

**Authors:** Neda Baniasadi, Franziska G. Rauscher, Dian Li, Mengyu Wang, Eun Young Choi, Hui Wang, Thomas Peschel, Kerstin Wirkner, Toralf Kirsten, Joachim Thiery, Christoph Engel, Markus Loeffler, Tobias Elze

**Affiliations:** 1 Leipzig Research Centre for Civilization Diseases (LIFE), Leipzig University, Leipzig, Germany; 2 Schepens Eye Research Institute, Harvard Medical School, Boston, MA, USA; 3 Institute for Medical Informatics, Statistics, and Epidemiology, Leipzig University, Leipzig, Germany; 4 Institute for Psychology and Behavior, Jilin University of Finance and Economics, Changchun, China; 5 Applied Computer Science and Biosciences, University of Applied Sciences Mittweida, Mittweida, Germany; 6 Institute of Laboratory Medicine, Clinical Chemistry and Molecular Diagnostics, Leipzig University, Leipzig, Germany

**Keywords:** retinal nerve fiber layer thickness, interocular, asymmetry, optical coherence tomography, glaucoma

## Abstract

**Purpose:**

The onset and progression of optic neuropathies like glaucoma often occurs asymmetrically between the two eyes of a patient. Interocular circumpapillary retinal nerve fiber layer thickness (cpRNFLT) differences could detect disease earlier. To apply such differences diagnostically, detailed location specific norms are necessary.

**Methods:**

Spectral-domain optical coherence tomography cpRNFLT circle scans from the population-based Leipzig Research Centre for Civilization Diseases–Adult study were selected. At each of the 768 radial scanning locations, normative interocular cpRNFLT difference distributions were calculated based on age and interocular radius difference.

**Results:**

A total of 8966 cpRNFLT scans of healthy eyes (4483 patients; 55% female; age range, 20–79 years) were selected. Global cpRNFLT average was 1.53 µm thicker in right eyes (*P* < 2.2 × 10^–16^). On 96% of the 768 locations, left minus right eye differences were significant (*P* < 0.05), varying between +11.6 µm (superonasal location) and −11.8 µm (nasal location). Increased age and difference in interocular scanning radii were associated with an increased mean and variance of interocular cpRNFLT difference at most retinal locations, apart from the area temporal to the inferior RNF bundle where cpRNFLT becomes more similar between eyes with age.

**Conclusions:**

We provide pointwise normative distributions of interocular cpRNFLT differences at an unprecedentedly high spatial resolution of 768 A-scans and reveal considerable location specific asymmetries as well as their associations with age and scanning radius differences between eyes.

**Translational Relevance:**

To facilitate clinical application, we implement these age- and radius-specific norms across all 768 locations in an open-source software to generate patient-specific normative color plots.

## Introduction

Retinal nerve fiber layer thickness (RNFLT) is an important adjunct to diagnosing optic neuropathies like glaucoma. Glaucoma is characterized by apoptosis of retinal ganglion cells and their axons, which leads to an increase in the size of the optic nerve cup, and consequent vision loss.^1^ The ability to detect changes in RNFL early in the course of glaucoma will lead to early diagnosis and treatment of the disease, which can improve the prognosis. Optical coherence tomography (OCT) is an established high-resolution imaging technology that objectively measures RNFLT.^2^^,^^3^ Common clinical ophthalmic OCT devices compare RNFLT with an age-matched normative database incorporated into the software for diagnostic purposes. Typical clinical RNFLT measurement protocols implemented in OCT devices measure thicknesses on a single circle with a diameter of about 3.5 mm around the optic nerve head (ONH).^4^ OCT devices provide printouts of RNFLT along the entire measurement circle together with percentiles of population norms. Thinning of the RNFL may occur at small areas on the measurement circle, which may be missed by summary measures such as coarse circle sectors. Therefore, owing to individual ocular anatomic variability, the onset of RNFL thinning may not fall outside normative limits if a patient's individual RNFLT geometry differs from normative expectations within each single eye.^5^^–^^7^

RNFLT is known to be associated with age and ocular magnification,^7^ race,^8^ and sex.^9^ Apart from that, it is commonly assumed that the RNFL geometries between the right and the left eye of healthy individuals are highly correlated. Because the onset of pathologic RNFL thinning is highly location specific, an interocular comparison of RNFLT may reveal further diagnostically relevant information in addition to the common practice of comparing either eye in isolation to population based norms. For instance, it has been shown that interocular RNFL asymmetry is a useful clinical and quantitative OCT measurement to assess early glaucomatous damage,^10^ and can be even helpful in differentiating subtypes of glaucoma.^11^ For instance, we have previously shown that the subtype of pseudoexfoliation glaucoma presents asymmetrically for most cases in clinic at the time of its diagnosis.^11^

**Figure 1.**
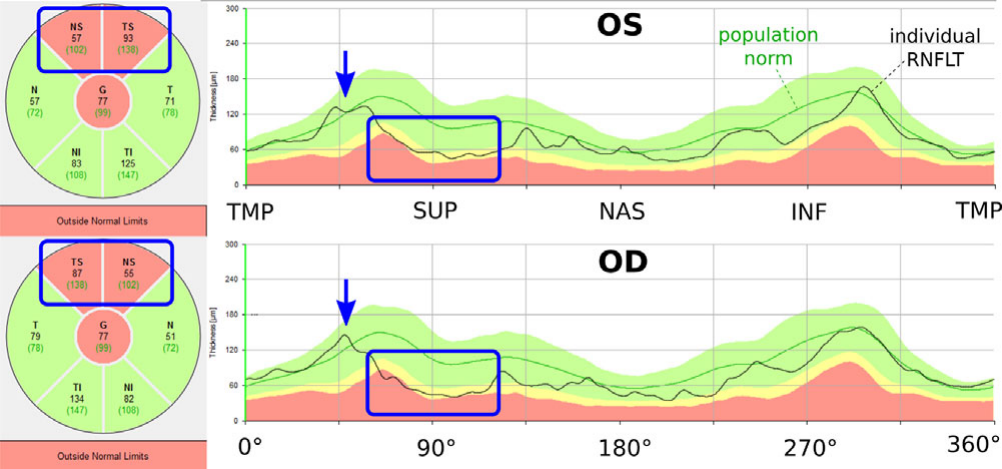
Illustrative printouts of the Spectralis RNFLT measurements of the left eye (OS) (*top*) and right eye (OD) (*bottom*) of a 28-year-old female (scanning radius difference between left and right eye: −19.8 µm) without glaucoma diagnosis, not taking interocular pressure reducing medication, and without any signs of an optic neuropathy on the fundus photographs, which are shown in Supplementary Figure S1. The individual major superotemporal RNFLT bundles of both eyes of the patient (*blue arrows*) are shifted in temporal direction compared with the population norm (*dark green line*). In each eye, nasal (NAS)–temporal (TMP) and superotemporal sectors are marked as outside normal limits by the machine, highlighted by *blue rectangles* in the figure. The RNFLT of each eye is marked as abnormal by the machine, and the individual RNFLT profiles of the two eyes (*black lines*) as well as the respective specific locations marked as abnormal look similar, which suggests a specific individual eye anatomy rather than an optic neuropathy as the reason for the abnormality marks. INF, inferior.

Interocular asymmetry may even help to interpret questionable RNFLT abnormality marks owing to individual eye anatomy deviating from the norm, as illustrated by an example in Figure 1 (see Supplementary Fig. S1 for fundus photographs of this patient). On the RNFLT measurements of either eye of this patient, the same sectors are marked as abnormally thin by the machine. However, the RNFLT profiles of the two eyes look similar. In particular, in both eyes of this individual, the superotemporal RNFLT maxima are shifted in a temporal direction compared with the machine norms. Although each eye individually deviates anatomically from the norm and is therefore flagged by the OCT software, the RNFLT difference between the eyes may still be within normal limits. To determine this, as well as to address the other points population statistics of interocular RNFLT differences along the entire measurement circle are needed. Although eyes are paired organs, numerous anatomic asymmetries have been described, even in ophthalmically healthy patients.^12^ Interocular RNFLT differences were studied on multiple occasions by time-domain and spectral-domain OCT over the past one and one-half decades.^1^^,^^13^^–^^16^ Consistently, these works reported significantly greater RNFLT values for right eyes. With respect to specific circumpapillary locations, previous works analyzed up to 12 RNFLT sectors. Although these works offer fundamental insight in interocular RNFLT geometry, they do not yet provide diagnostically applicable normative distributions over the full circumpapillary OCT scanning circle, that is, the measurement protocol that is frequently applied in clinical practice to support glaucoma diagnoses.

In the current study, we investigate the following: (1) circumpapillary interocular RNFLT differences at a spatial resolution of 768 samples; (2) the impact of age as well as the true radius of the circumpapillary scanning circle, which related to individual ocular anatomy, on interocular RNFLT differences (as previously been shown to be strongly associated with RNFLT^7^; and (3) the impact of individual RNF bundle geometry, and the location of major RNFLT peaks on interocular RNFLT differences. The final outcomes of this work are age-, radius-, and location-specific normative distributions of circumpapillary interocular RNFLT differences with a spatial resolution of 768 locations. To facilitate the application of these norms in ophthalmic research and their incorporation into OCT devices for future clinical use, we provide the entire distributions numerically in an open access repository, at http://dx.doi.org/10.17632/ss2nynfc8s.1, together with an easy-to-use R function, which graphically displays the measurement curve for RNFLT with the respective normative limits.

The current investigation therefore presents the first interocular comparison leading to a normative database. Contrary to previous investigations, our study presents percentile data, enabling the detection of individual thickness measurements out of the norm. In particular, this strategy makes it possible to graphically illustrate the RNFLT circle scan superimposed on colored normative percentiles very similar to the thickness color plots that are part of the standard printouts of most clinical OCT devices, which provides clinicians with a graphical representation they are familiar with. Furthermore, it enables precise quantitative analyses of subtypes of glaucoma and other optic neuropathies with regard to their respective interocular asymmetry compared with healthy controls, which may improve the early detection of pathologic RNFL thinning.

## Methods

### Participant Population

Participants of this study are selected from the baseline assessment of a large population-based cohort study conducted by the Leipzig Research Centre for Civilization Diseases (LIFE Adult Study) at Leipzig University, from August 2011 to November 2014. LIFE Adult includes 10,000 randomly selected participants from just over one-half of a million inhabitants of Leipzig, a city located in the east of Germany. All participants provided informed consent to participate. The institutional ethics board of the Medical Faculty of Leipzig University approved the study and the research followed the Declaration of Helsinki.

The recruitment of LIFE Adult patients occurred in an age- and gender-stratified manner, with focus on the ages between 40 and 80 years. More specifically, in total, 400 patients between 19 and less than 40 years of age and 9600 patients between 40 and less than 80 years of age were recruited. Recruitment targeted to balance each decade within these two age groups with respect to number of patients and gender. The six 19-year-old patients were categorized into the 20 to 30 decade for this purpose.

### Data Collection

All participants underwent an extensive assessment, including structured interviews, questionnaires, physical examinations, and blood and urine sample collections. Ophthalmologic imaging included OCT (Spectralis, Heidelberg Engineering, Heidelberg, Germany; Spectralis HRA + OCT, Aquisition Module 5.4.7.0), and undilated fundus images (Nidek AFC-230). OCT was performed to obtain circumpapillary RNFLT (cpRNFLT) scans with a resolution of 768 equidistant measurement points placed on a circle around the ONH. The circle location and its coordinate system are illustrated in Supplementary Figure S2. More details regarding LIFE Adult Study data can be found in our recently published articles.^7^^,^^17^ For the current analysis, the 10,000 baseline participants of the LIFE Adult project underwent the following exclusion process, as illustrated by the flowchart in Supplementary Figure S3.

During the early stages of the LIFE Adult study, the Spectralis SD-OCT machine was out of service owing to a hardware defect. Therefore, OCT imaging could not be performed until the device was repaired. The hardware failure also affected the database of existing scans, so that several measurements of some patients or single eyes were partially damaged and could not be exported from the machine for data analysis.

Of the remaining 17,974 eyes of 9069 participants, 2678 patients were excluded based on clinically significant findings on ophthalmic imaging. For this, two independent, experienced and clinically trained observers analyzed OCT scans and fundus images. In case of interobserver differences, a consensus decision was reached to classify the participant's eye. Clinical and subclinical ophthalmic findings were graded based on current ophthalmologic standards. Participants with clinical disease of the posterior eye within macula and optic nerve regions of at least one eye were excluded from the current study: all cases of retinal detachment or retinal hole, retinal pigment epithelium detachment, edema, bleeding, vascular abnormalities (such as vascular occlusion, ischemia, retinal vascular tortuosity, aneurysm, neovascularization), any kind of scarring, atrophy, fundus with disseminated white areas, cotton wool spots, and fibrosis if traction or puckering with foveal involvement was observed. A patient was also excluded if a tumor was present or a staphyloma was detected. For the ONH specifically, patients were furthermore excluded who presented with the following in at least one eye: suspected glaucoma, violation of ISNT rule, vertically oval with a cup-to-disc ratio of greater than 0.7, optic disc pit, coloboma of the optic disc, bleeding, neovascularization, optic atrophy, sectorial paleness, swelling of the ONH, papilledema, and optic disc drusen. Within the macular region in specific the following additional exclusion applied: age-related macular degeneration stages 3 and 4, and maculopathy unrelated to age-related macular degeneration (stage 5), previously described by the Gutenberg Health Study^18^ and based on the Rotterdam classification.^19^^,^^20^

Furthermore, patients were excluded if they reported a glaucoma diagnosis in the medical interview and/or if an intraocular pressure–lowering medication was present among all medications the patient took at the time of the test.

Only images with reliable measurements for both eyes were included in data analysis. Reliability criteria included (1) image quality of 20 dB or greater, (2) an average number of B-scans of 50 or greater, and (3) no more than 2.5% missing or unreliable cpRNFLT segmentations among the 768 A-scans.

Details of inclusion and exclusion and the final sample included into the current analysis can be found in Supplementary Figure S3.

For all data analyses, left and right eyes were represented in a normalized coordinate system which starts at the temporal pole, proceeds to the superior, then nasal, then inferior pole, and finally ends up at the temporal location again, regardless of eye laterality.

### Statistical Analysis

All statistical analyses were performed using R programming language (version 3.5.2)^21^ and the GAMLSS R library.^22^

Paired *t*-tests with adjustment for multiple comparison by the Bonferroni method were calculated for the RNFLT differences between left and right eyes for global RNFLT as well as separately for each of the 768 retinal locations on the OCT circle scan. Furthermore, because it has previously been shown that ocular magnification (typically parameterized by axial length/ refractive error, corneal refractive power or scanning focus) has an impact on RNFLT measurements,^6^^,^^7^^,^^23^^–^^25^ we aimed to assess the true radius of the circle scan of each eye. The Spectralis OCT machine projects a scanning circle of a fixed radius of 6° into the eye, which results in different circle sizes on the retina, depending on ocular magnification. Therefore, Spectralis OCT uses the focus settings adjusted by the device operator to estimate the true scanning radius on the retina according to a widely applied model.^26^ Radius differences between the two eyes, for instance owing to axial length or corneal refractive power, are likely to affect interocular RNFLT symmetry, because RNFLT is then measured at different retinal locations in either eye.

We tested the influences of radius difference (left minus right eye), absolute radius difference, and age for each of the 768 retinal locations on the circle. More specifically, we first calculated linear models of the mean and variance of left minus right eye RNFLT differences with age and absolute radius difference as regressors. For each retinal location, a model comparison based on the Akaike information criterion^27^ determined possible impacts of age, absolute radius difference, or their combination on mean and variance (normal distribution). Second, because the results indicated several nonlinear effects for specific locations (see the Results section), we calculated a comprehensive Akaike information criterion–based model comparison with 12 parameters for each retinal location, namely a third-degree polynomial of age and a third-degree polynomial of radius difference for mean and variance, respectively, which implied the comparison of 2^12^ = 4096 models at each of the 768 locations. In other words, we computationally determined the optimal normative distributions of interocular RNFLT differences depending on age and interocular radius difference separately for each retinal location. As the final outcome, we receive a single function that returns a normative distribution depending on the following three parameters: retinal location (in degree around the ONH), age, and interocular radius difference. More comprehensive details on the implementation can be found in the Supplementary Material.

On the temporal retina, nerve fiber axons shape two major bundles, as schematically illustrated on Supplementary Figure S2. On the measurement circle, the locations of these bundles are visible as the two areas of thickest RNFL. As illustrated in Figure 1, individual locations of these two major RNFLT humps, that is, the superotemporal hump (blue arrow in Fig. 1) and the inferotemporal hump (the equivalent hump on the inferior retina, visible as the second local RNFLT maximum in Fig. 1) may considerably vary among individuals, as demonstrated by the histograms in Supplementary Figure S4, but their symmetry among the left and right eyes has, to our best knowledge, not been investigated before. Therefore, we additionally studied the relative difference of the major superotemporal and inferotemporal RNFLT peaks between right and left eyes. In particular, we smoothed the RNFLT measurements of each eye by a moving average of ±12° around each location to remove the effect of random fluctuations. Superotemporal and inferotemporal peaks were then determined as the local RNFLT maxima of superior respectively inferior retina. The impact of age and radius differences on the peak differences was determined by linear regression.

## Results

**Figure 2.**
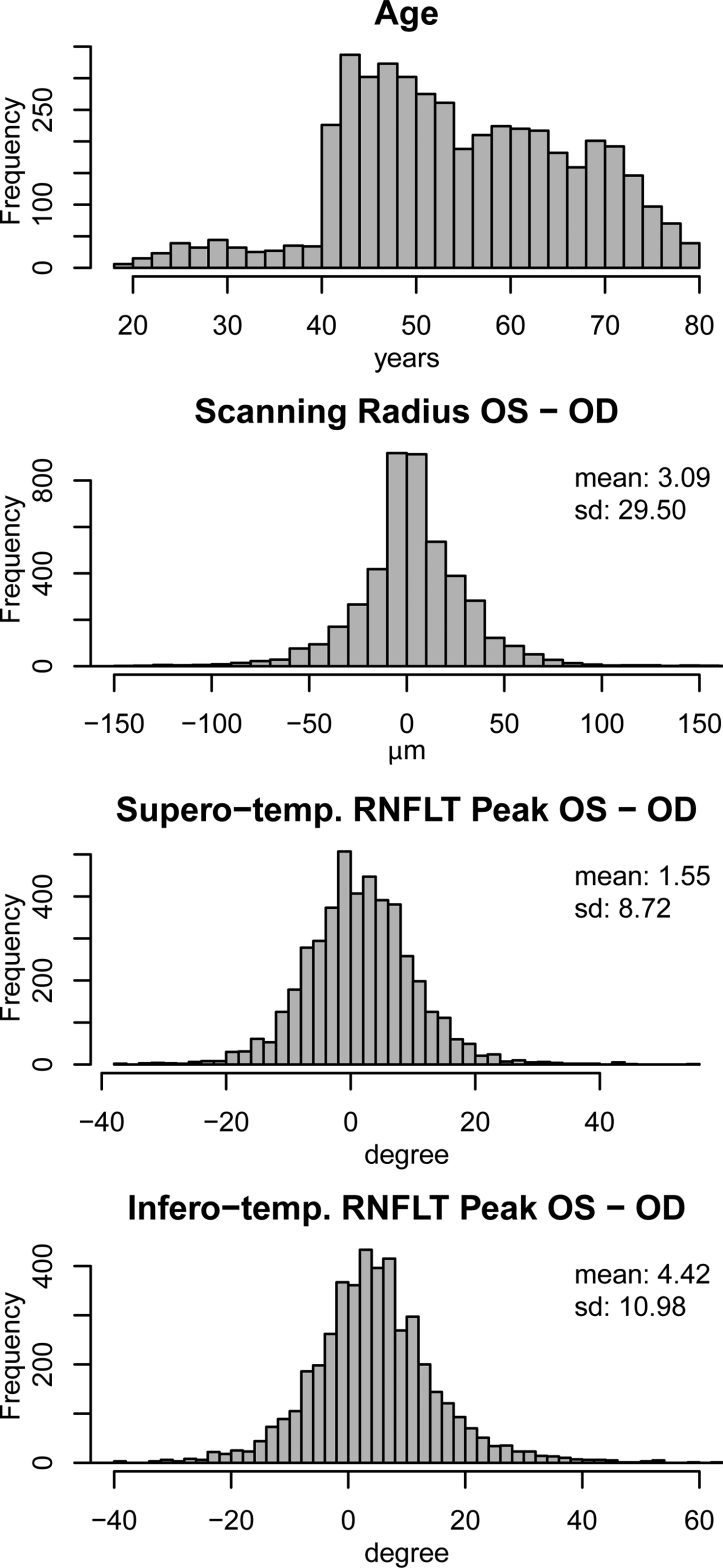
From top to bottom: Histograms of age, scanning radius difference, and difference between superotemporal and inferotemporal RNFLT peaks between left (OS) and right eyes (OD).

**Figure 3.**
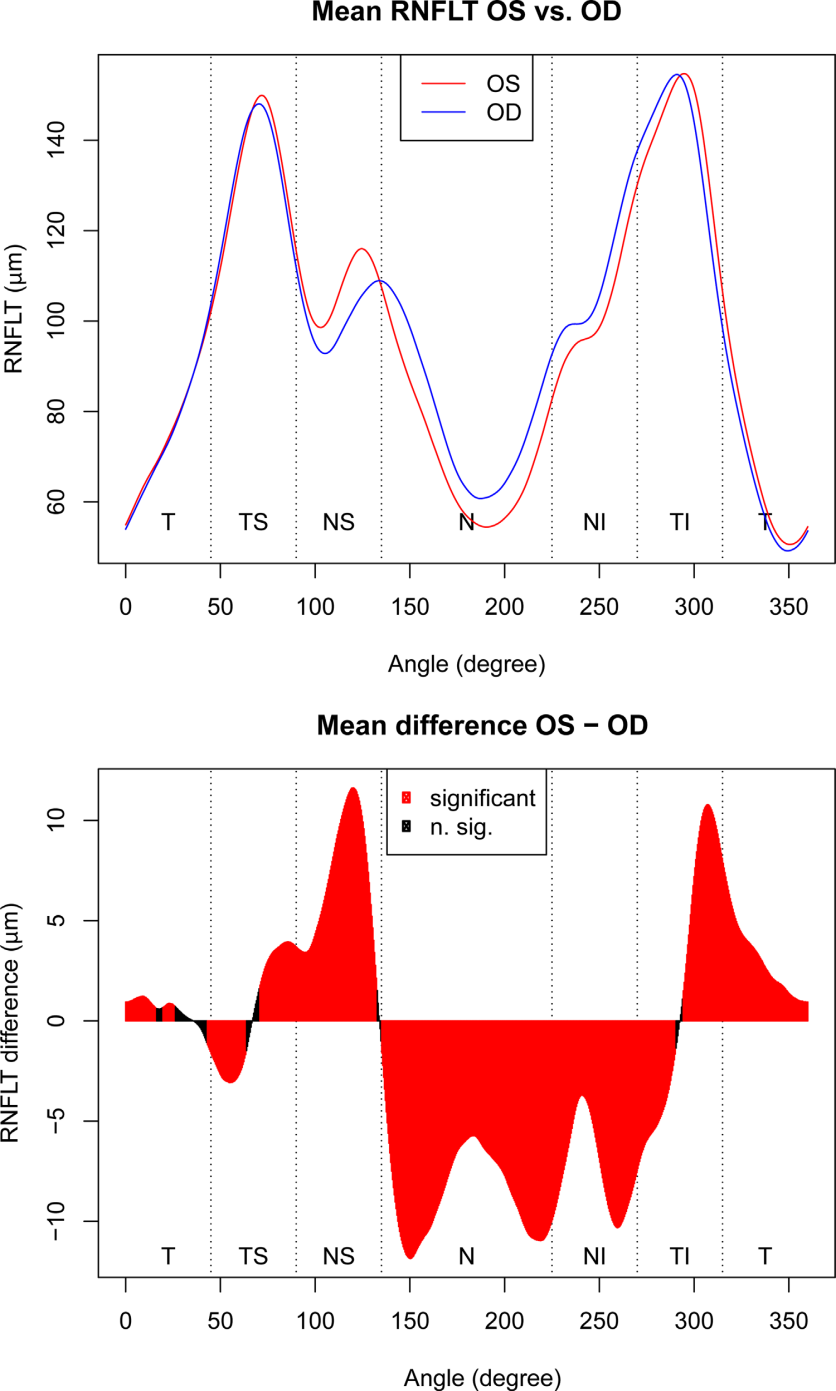
Mean RNFLT separately for left (OS) and right (OD) eyes (*top*) and mean RNFLT differences (*bottom*), for each of the 768 locations around the scanning circle, respectively. The coordinate system starts at the temporal (T) pole (0°) and traverses clockwise to the superior (S), nasal (N), inferior (I) and back to temporal locations. In the difference plot, red locations denote significant (*P* < 0.05) RNFLT differences between the eyes, calculated by paired *t*-tests on each location and adjusted for multiple comparisons.

There were 8966 RNFLT scans of 8966 eyes of 4483 patients (2466 female and 2017 male) that were selected based on our inclusion criteria. Supplementary Table S5 shows the demographic information for our study population. Figure 2 shows, from top to bottom, the histograms of age and scanning radius difference, as well as the difference between superotemporal and inferotemporal RNFLT peaks between the left and right eyes. The average scanning radius of right eyes was 3.09 µm smaller compared with left eyes (*t*-test, *P* = 2.5 × 10^−^^12^). The RNFLT average over all 768 locations was 1.53 µm thicker in right eyes compared with left eyes (*t*-test, *P* < 2.2 × 10^−^^16^). The interocular difference varied substantially over the retinal locations, as illustrated in Figure 3, which shows pointwise mean RNFLT separately for right and left eyes (top) as well as the according pointwise mean RNFLT differences between the eyes (bottom). On 95.6% of the locations, differences were significant (*P* < 0.05, red color) after adjustment for multiple comparisons over the 768 scanning locations. The differences vary between +11.6 µm (superonasal location) and –11.8 µm (nasal location). The figure further illustrates the substantial location specificity of the effects: Although for most of the nasal area, the RNFL is significantly thicker in right eyes, for superior and inferotemporal areas, the RNFL is thicker in left eyes. Supplementary Figure S6 shows histogram of the mean absolute RNFLT differences, averaged over all 768 locations.

RNFLT peak locations significantly differ between the eyes: Whereas the superotemporal peaks of left eyes are 1.55° more nasal than the peaks of right eyes (*t*-test, *P* < 2.2 × 10^−^^16^), the inferotemporal peaks of right eyes are 4.42° more nasal than those of left eyes (*t*-test, *P* < 2.2 × 10^−^^16^). The interpeak angle (i.e., the angle between the two peaks, as illustrated in Supplementary Fig. S2) across eyes are 2.86° larger in right eyes (*t*-test, *P* < 2.2 × 10^−^^16^). Although the interpeak angle difference was weakly but significantly inversely correlated with the radius difference (Pearson's r = −0.08, *P* = 5.9 × 10^−^^8^), differences in radius explain only 0.65% of the variance of the differences in interpeak angle (by analysis of variance). Linear regression with mean interpeak angle between the two eyes as regressor and superior/inferior peaks as dependent variable yielded a significant positive association between interpeak angle and inferior (β = 0.05, *P* = 1.26 × 10^−^^7^) but not superior peak difference (β = 0.01, *P* = 0.12).

**Figure 4.**
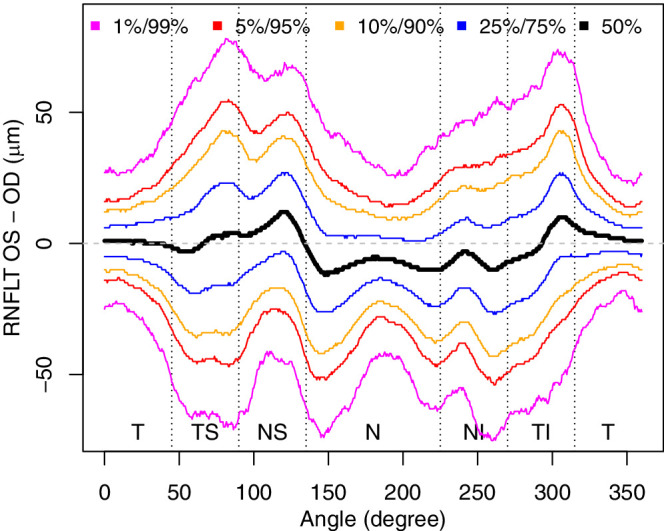
Sample quantiles of RNFLT differences between left (OS) and right (OD) eyes for each of the 768 locations around the scanning circle, over all patients, regardless of age or scanning radius. SD, standard deviation.

Figure 4 illustrates the empirical quantiles of interocular RNFLT differences for each of the 768 retinal locations around the measurement circle, regardless of age and scanning radius difference. Not only the mean of the difference, but also the variance vary substantially over different retinal locations, with the greatest dispersions around the locations of the two major nerve fiber bundles and smallest dispersions at temporal locations.

Supplementary Figure S7 shows on which circumpapillary locations age and absolute scanning radius difference between right and left eye have an impact (lower Akaike information criterion compared with null model) on the mean or variance of interocular RNFLT differences. The mean and the variance increase with increasing age and absolute radius difference particularly around the location of the major superior RNFLT peak. Advancing age is also associated with larger RNFLT differences and increasing variance nasal to the major inferior RNFLT peak. Temporal to this peak, however, advancing age is related to a smaller mean interocular RNFLT differences and variances, that is, the left and right RNFL geometry becomes more similar over age in this region.

## Discussion

Our study shows that the average RNFLT value over all 768 locations was 1.53µm greater in right eyes compared with left eyes (t-test, *P* < 2.2 × 10^−^^16^). The interocular difference varied substantially over the retinal locations, as illustrated in Figure 3. Interocular RNFLT differences are significant in over 95.6% of the locations on ONH circle scan (*P* < 0.05, red color) after adjustment for multiple comparisons. The differences vary between +11.6 µm and –11.8 µm. Our study also demonstrates that RNFLT values were significantly greater in superior and inferotemporal areas in left eyes compared with right eyes.

**Figure 5.**
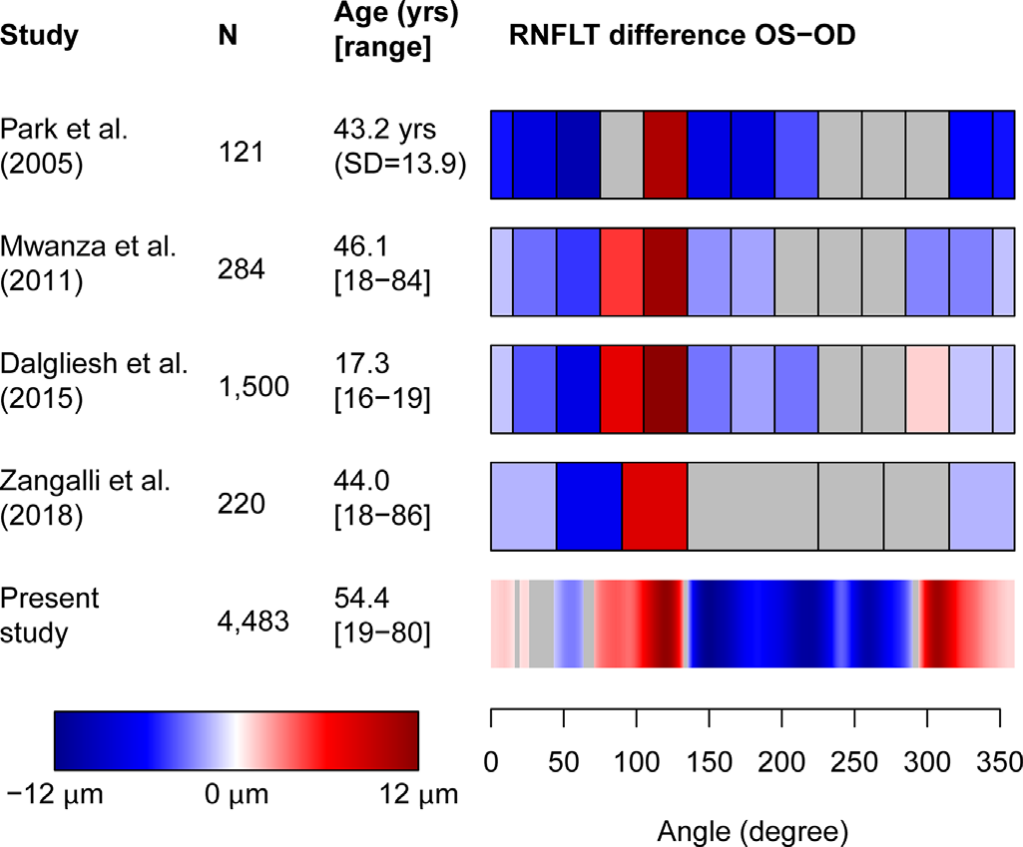
Comparison with previous studies of interocular RNFLT differences. Previous studies with sample size N greater than 100 were selected if they applied OCT to measure RNFLT and were not restricted to children as participants. The Age column shows mean and range (apart from the Park et al. study, which did not report an age range). *Blue/red color* denotes significantly thicker RNFL in right/left eyes, respectively, for each circumpapillary sector or location. Gray color denotes the lack of significant differences. Note that each temporal sector of the previous works was split, with each half of the sector displayed on the right and left ends of the bar, respectively, to align the coordinates with our study.

The results of this study were compared with previous studies, which measured interocular RNFLT differences (Fig. 5). Although previous studies (top four rows) provided results for 6^13^ to at most 12 sectors,^14^^–^^16^ our data (bottom row) offer a spatial resolution of 768. All previous studies had sample sizes between 121 and 1500, which is substantially lower than our sample size of 4483. Previous results cover one time-domain (Stratus OCT; Park et al.^16^) and two different spectral-domain OCT machines (Cirrus HD-OCT: Mwanza et al.,^15^ Dalgliesh et al.^14^; Spectralis OCT: Zangalli et al.).^13^ Gray sectors denote the lack of significant results, which occurred in all previous studies at between 16.7% and 50.0% of the measurement circle, especially in inferonasal sectors. Because null hypothesis significance testing does not allow to test for equality, the absence of significant effects might either mean that there are indeed no differences at these locations, or that the variance on these locations is too high and the number of patients too low to identify the direction of the effect. In the present study, only 9.2% of the locations do not exhibit significant difference effects, and these locations are typically arranged around the reversal points between positive and negative differences, which suggests that both the larger sample size and the large number of measurement locations in our study considerably decrease the uncertainty of interocular asymmetry effects. Our work reveals a distinct region of thicker RNFL in left eyes in the superior area, which is generally consistent with previous studies, but our high spatial resolution allows a more precise characterization and localization of its reversal points. In addition, our results show a second such region in the inferotemporal area, which is only visible in one of the previous studies.^14^

The RNFLT line graph (top panel of Fig. 3) indicates two different reasons for the obtained interocular differences: First, at certain retinal locations, the RNFL truly differs in thickness between the left and right eyes. This is visible in most of the superior retina, where the red curve is above the blue curve, which means in this location left RNFL is thicker than right RNFL, as well as on the entire nasal and inferonasal areas, where the blue curve is above the red curve, which means left eyes have thinner RNFL in this area compared with right eyes. In the inferotemporal area, however, the two curves indicate that the thickness difference is mainly driven by the location difference between the two equally high RNFLT peaks, that is, the major nerve fiber bundle in the left eye, although equally thick, is shifted in temporal direction compared with the corresponding bundle in the right eye. This finding implies that the inferotemporal hump in the bottom panel of Figure 3 is a result of a phase shift between the two eyes, that is, of different interocular RNF geometry, rather than of a true point-wise thickness difference. This finding is consistent with our finding that inferotemporal peaks of right eyes are 4.4° more nasal than those of left eyes. Because refractive error is closely related to the true diameter of the scanning circle, which has a considerable impact on the RNFLT profile,^7^ and scanning circle differences are associated with shifts in RNFLT humps,^6^^,^^23^^,^^28^ one might assume that anisometropia is a major determinant of the reversal locations of the interocular RNFLT asymmetries. However, our results suggest this scenario to be unlikely, because scanning radius effects account for only less than 1% of the variance of the differences in interpeak angle.

Ocular dominance is subject to a considerable right eye preference,^29^^,^^30^ which is an interesting coincidence with our findings of thicker RNFL in right eyes on average. Choi et al.,^31^ in agreement with our study, reported globally thicker circumpapillary RNFL in right eyes, which, however, could not be explained by dominance effects alone; however, in dominant eyes, the inferior RNFL was thicker than the superior RNFL. Our substantially more detailed ocular asymmetry analysis might help future studies to investigate the possible associations between RNFLT differences and ocular dominance more comprehensively.

Interocular RNFLT differences vary over retinal locations not only in mean but also considerably in variance, as illustrated in Figure 4 and Supplementary Figure S7. The variance is particularly large around the locations of the two major RNFLT humps, that is, at retinal areas of high relevance for glaucoma diagnostics. Furthermore, age and the interocular difference of the estimated true scanning circle radius (which is, as discussed elsewhere in this article, related to anisometropia) significantly modulate both mean and variance of interocular RNFLT differences over large retinal areas (Supplementary Fig. S7): Although larger radius differences typically increase the magnitude and variance of interocular asymmetries (red areas), this is not necessarily the case for increasing age, because there are certain, distinct circumpapillary regions where the RNFLT becomes more similar between the eyes with aging (blue areas). These findings justify our approach to independently model mean and dispersion of the normative RNFLT difference distributions separately for each of the 768 circumpapillary locations as functions of age and the (signed) interocular difference of the estimated true scanning radius.

Details of the generation of the interocular RNFLT difference norms are provided as supplementary material in the external data repository, and the accompanying numerical values on which the age, radius, and location specific norms are based are provided in a public open access data repository. The supplemental software allows to generate normative plots for any age and scanning radius differences. For instance, Supplementary Figure S8 illustrates selected percentiles for two exemplary cases, namely, for 50-year-olds with equal scanning radii in both eyes (top panel) and for 70-year-olds with a radius difference of 50 µm.

**Figure 6.**
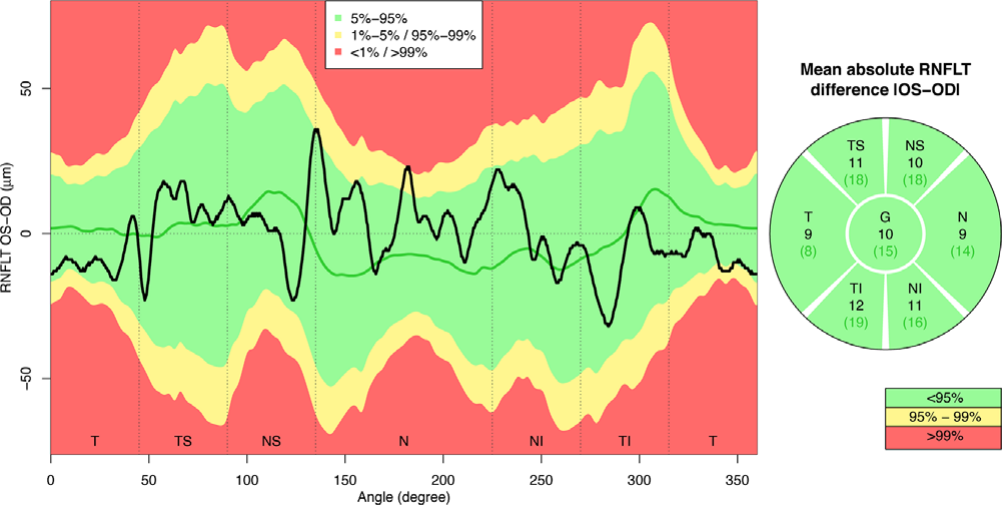
Example of the interocular difference normative plot generated by our supplemental software provided in the external supplementary data repository. The measurement shown here, together with the corresponding personalized norms according to age and scanning radius, is the same as shown in Figure 1. Colors and layout correspond with the Spectralis printout (Fig. 1). (*Left*) The interocular RNFLT difference (left minus right eye; *solid black line*) is shown together with the personalized normative quantiles. The *dark green line* shows the normative median. On the right, the absolute interocular RNFLT difference for the sectors as well as the global mean (G) are presented (*black numbers*), together with the respective normative medians (*dark green numbers in parentheses*).

To facilitate applications in basic and medical research and to support future implementations of our results by OCT manufacturers, in the supplementary data repository, we provide open source software that takes interocular RNFLT differences as input and generates plots inspired by the Spectralis standard printout, displaying the interocular difference curve on the top of the patient's personalized normative percentile bands, together with details of the major sectors. To facilitate practical applications, layout and coloring corresponds with conventions used by typical clinical ophthalmic OCT devices, such as the Heidelberg Spectralis or Cirrus HD-OCT (Carl Zeiss Meditec, Jena, Germany). Figure 6 shows the result of the application of our software to the measurement shown in Figure 1. The layout and colors of the graphical output correspond with the Spectralis printout, but show the interocular RNFLT difference together with the corresponding personalized norms according to age and scanning radius.

We would like to note that the normative plots generated by our provided software are descriptive and do not imply precise details on how to interpret possible deviations from these norms within a specific eye. Rather, they should be used in a similar way as the existing color plots for RNFLT currently provided by OCT manufacturers, that is, as a visually inspected adjunct to the complex diagnostic decision process. As with the RNFLT, small and localized deviations from RNFLT difference norms do not necessarily indicate ocular disease, and further research might reveal particularly more or less informative circumpapillary regions of deviations from RNFLT difference norms, which help to better understand and interpret details of the plots we provide here.

Limitations of this study include that only one OCT machine (Spectralis SD-OCT) was used to obtain the normative distributions and that the population was largely dominated by European patients; therefore, it remains to be established if our results can be generalized to people of other descent. Further studies are needed to investigate this in detail. However, we would like to note that the results from previous studies summarized in Figure 5 are based on several different OCT machines and include a variety of non-European populations, and their sectoral results share several distinct features with our study, as detailed elsewhere in this article. A further potential deficiency of the dataset we used for this study is the unavailability of axial length, refractive error, and corneal refractive power. The availability of axial length and corneal refractive power might allow a more accurate and more established estimate of ocular magnification. At the same time, we would like to note that none of the OCT machines currently used in clinical practice include refractive error data in any of their norms, and thus we based our interocular difference norms only on data readily available in the Spectralis machine, which is the case for both age and the estimated scanning radius. Refractive error might indeed modulate interocular RNFLT differences. Therefore, as detailed elsewhere in this article, we systematically investigated those retinal characteristics that are known to be closely associated with refractive error, namely, the estimated true scanning radius, which depends on both lens related characteristics and axial length, and the location of the major RNFLT humps, which is correlated with axial length.

Finally, we would like to note that our study focused only on those RNFLT-related parameters that are established in current norms or are at least easily available from currently applied clinical scanning protocols, so that our results can be straightforwardly applied to existing OCT measurements from clinical practice. However, a number of further parameters, which are more complex to access, might additionally have an impact on RNFL thickness and therefore also on interocular RNFLT asymmetry. For instance, although we controlled for ocular magnification, there might be asymmetries in the true size of the optic disc, which, in turn, might affect RNFLT differently in the two eyes. Apart from that, even after normalizing the scanning coordinate system for the disc–fovea angle, existing asymmetries in the disc–fovea angle might have an impact on RNFLT asymmetry. Moreover, our results indicate impacts of individual RNFLT humps on interocular asymmetries, so that a precise knowledge of predisease RNF bundle locations in each eye would likely help to improve glaucoma diagnostics based on RNFLT asymmetry. In clinical settings, at the time of the first test, RNFL thinning may already have taken place owing to optic neuropathies, so that the predisease RNF bundle geometry cannot be determined anymore. To overcome this problem, it has been previously suggested to use the locations of the major retinal arteries, which are correlated with RNFLT peaks, as estimates of predisease RNF bundle trajectories. The relationship between vessel anatomy and RNFLT has been extensively studied.^5^^,^^24^ In a previous work, for instance, we could show that individual major retinal artery locations biased OCT RNFLT abnormality patterns on 37% of the peripapillary scanning area, regardless of glaucoma severity.^24^ Therefore, it is likely that interocular asymmetry–based glaucoma diagnostics could considerably be improved by taking individual vessel anatomy into account. This process, however, would require an initial automated artery detection on OCT fundus images. Future studies would be useful to implement these types of image processing and, in succession, to systematically study vascular anatomy on RNFLT asymmetry.

To conclude, we systematically investigated interocular differences in cpRNFLT and possible associations with age, scanning radius, and nerve fiber bundle geometry, in a large population-based study. As a final outcome, we provide detailed, high-resolution normative data specific to circumpapillary retinal location, age, and the difference of the scanning radii between the eyes. To facilitate applications, the normative distribution data as well as a software that takes cpRNFLT measurements as input and generates personalized normative color plots inspired by the Spectralis printout are provided in the open access repository.

Supplementary data and software can be found in the Mendeley repository linked with this article at http://dx.doi.org/10.17632/ss2nynfc8s.1. Supplementary Figures S1, S2, S3, S4, Supplementary Table S5, and Supplementary Figures S6, S7, and S8 are available under the Supplements menu.

## Supplementary Material

Supplement 1

Supplement 2

Supplement 3

Supplement 4

Supplement 5

Supplement 6

Supplement 7

Supplement 8
