# Supplementary figures and images for: Norms of Interocular Circumpapillary Retinal Nerve Fiber Layer Thickness Differences at 768 Retinal Locations
Source: Transl Vis Sci Technol. 2020 Aug 12;9(9):23. doi: 10.1167/tvst.9.9.23 (PMC7442876; doi:10.1167/tvst.9.9.23)

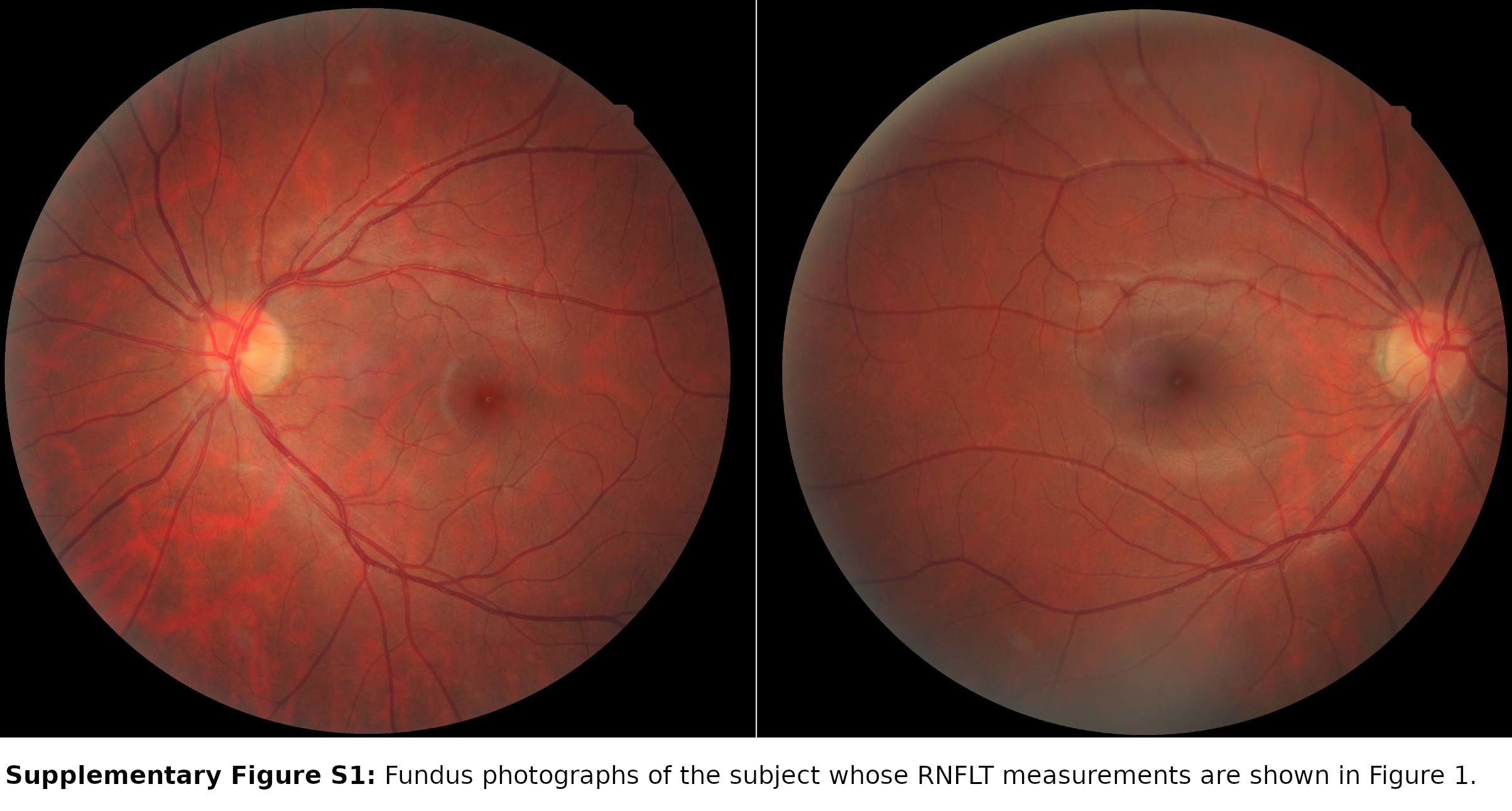

Supplement: Supplement 1 [file tvst-9-9-23_s001.jpg]

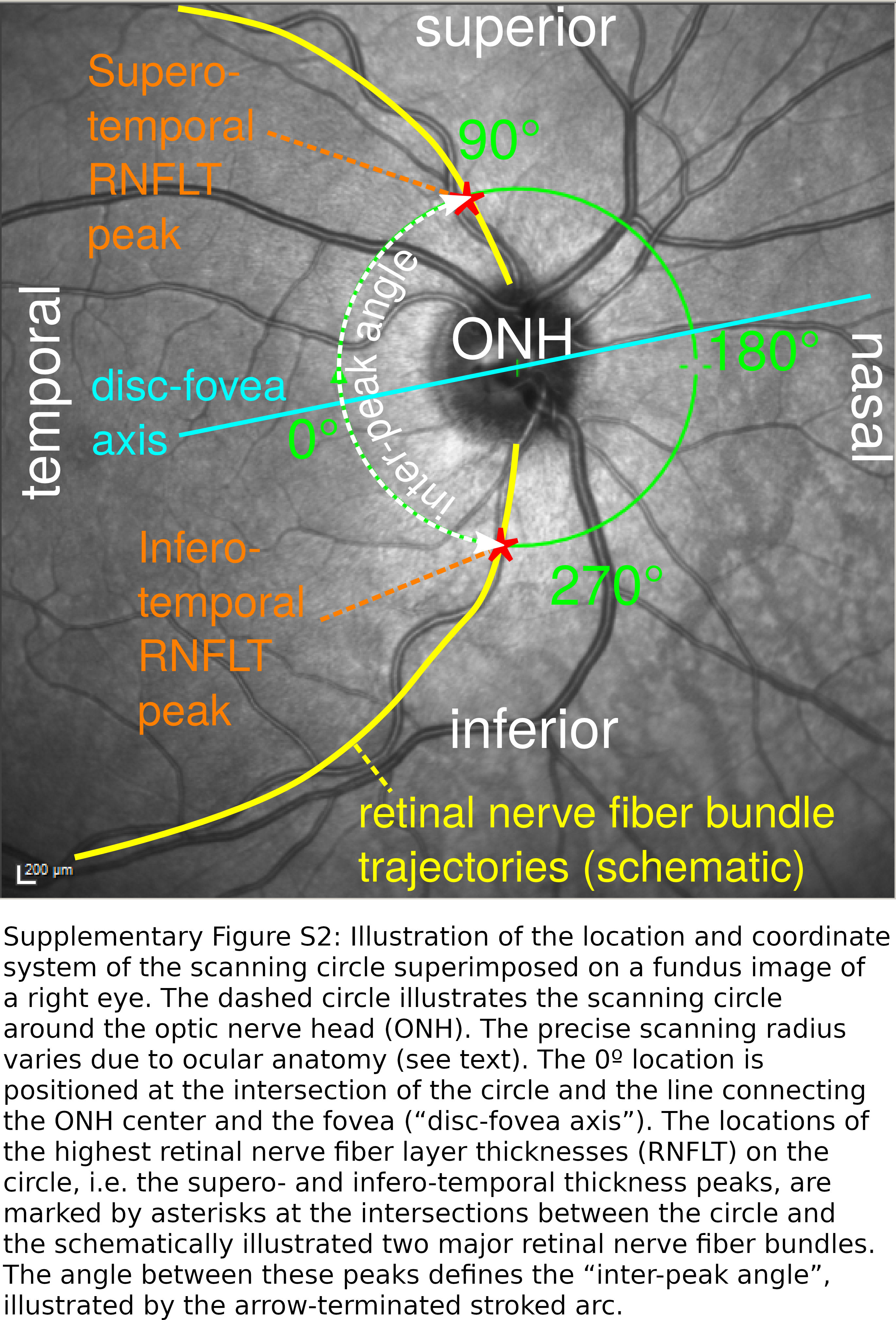

Supplement: Supplement 2 [file tvst-9-9-23_s002.jpg]

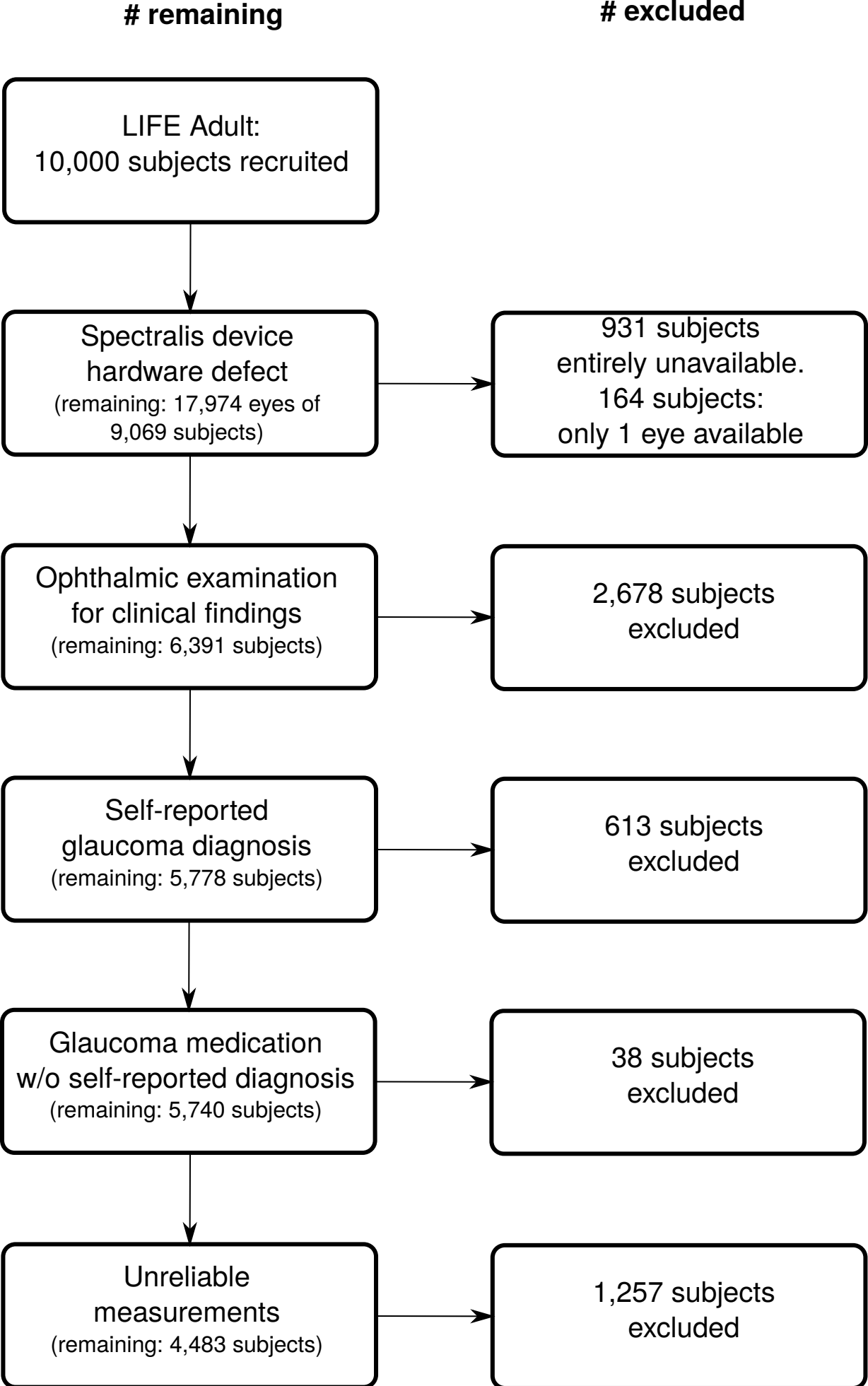

**Supplementary Figure S3:** Subjects inclusion and exclusion process.

Supplement: Supplement 3 [file tvst-9-9-23_s003.pdf]
