# Supplementary material for: Norms of Interocular Circumpapillary Retinal Nerve Fiber Layer Thickness Differences at 768 Retinal Locations
Source: Transl Vis Sci Technol. 2020 Aug 12;9(9):23. doi: 10.1167/tvst.9.9.23 (PMC7442876; doi:10.1167/tvst.9.9.23)
Supplement: Supplement 5 [file tvst-9-9-23_s005.pdf]

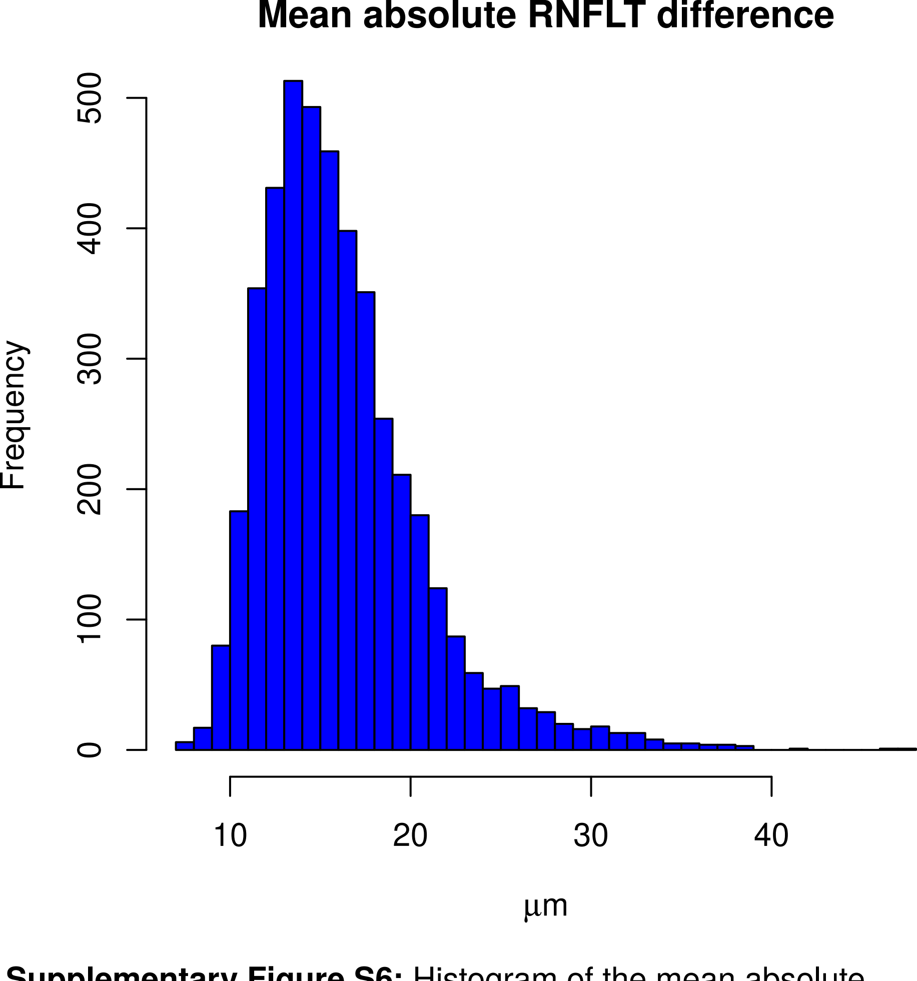

Supplementary Figure S6: Histogram of the mean absolute RNFLT differences, averaged over all 768 locations.
