# Supplementary material for: Norms of Interocular Circumpapillary Retinal Nerve Fiber Layer Thickness Differences at 768 Retinal Locations
Source: Transl Vis Sci Technol. 2020 Aug 12;9(9):23. doi: 10.1167/tvst.9.9.23 (PMC7442876; doi:10.1167/tvst.9.9.23)
Supplement: Supplement 6 [file tvst-9-9-23_s006.pdf]

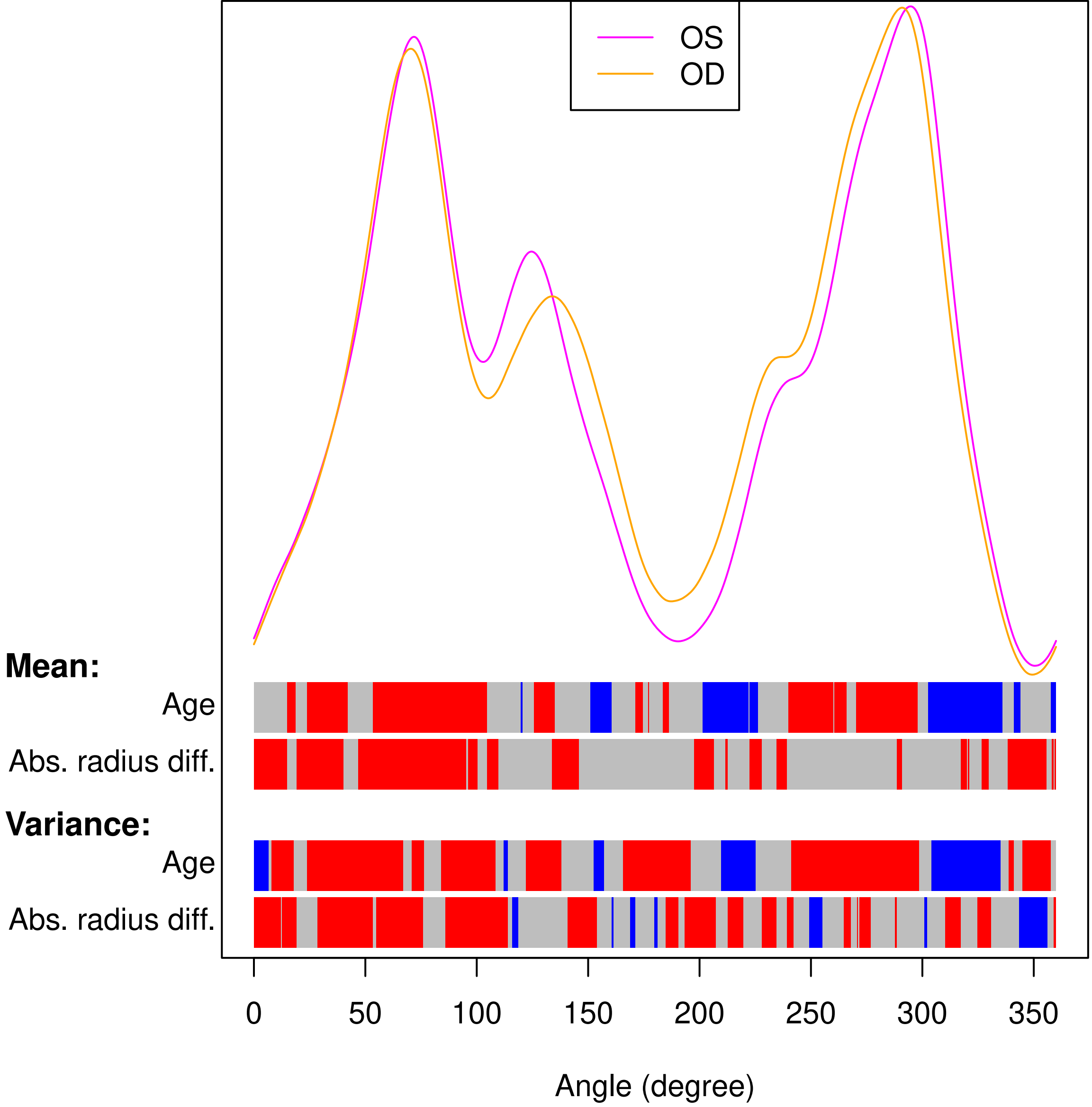

Supplementary Figure S7: Effects of age and absolute scanning radius difference between the two eyes on mean and variance of interocular RNFLT differences at each of the 768 retinal locations around the optic nerve head. On the top, average RNFLT curves are shown, separately for right and left eyes, as a spatial reference. Red/Blue locations denote that increasing parameter values are related to an increase/decrease of mean or variance of interocular RNFLT differences (lower AIC compared to the "null model" of intercept alone for each parameter). No effects were found for locations in gray. To determine the impacts of age and radius difference, linear regression models have been fitted to mean and variance of the RNFLT difference distributions at each of the 768 circumpapillary locations.

Note that the red and blue colors only denote the direction of the association (red: positive association, blue: negative association). The exact values of the fitted coefficients can be found in the supplementary table rnflt_difference_norms.csv which is available as a file and part of the Supplementary data and software repository (see below).
