# Supplementary material for: Norms of Interocular Circumpapillary Retinal Nerve Fiber Layer Thickness Differences at 768 Retinal Locations
Source: Transl Vis Sci Technol. 2020 Aug 12;9(9):23. doi: 10.1167/tvst.9.9.23 (PMC7442876; doi:10.1167/tvst.9.9.23)
Supplement: Supplement 7 [file tvst-9-9-23_s007.pdf]

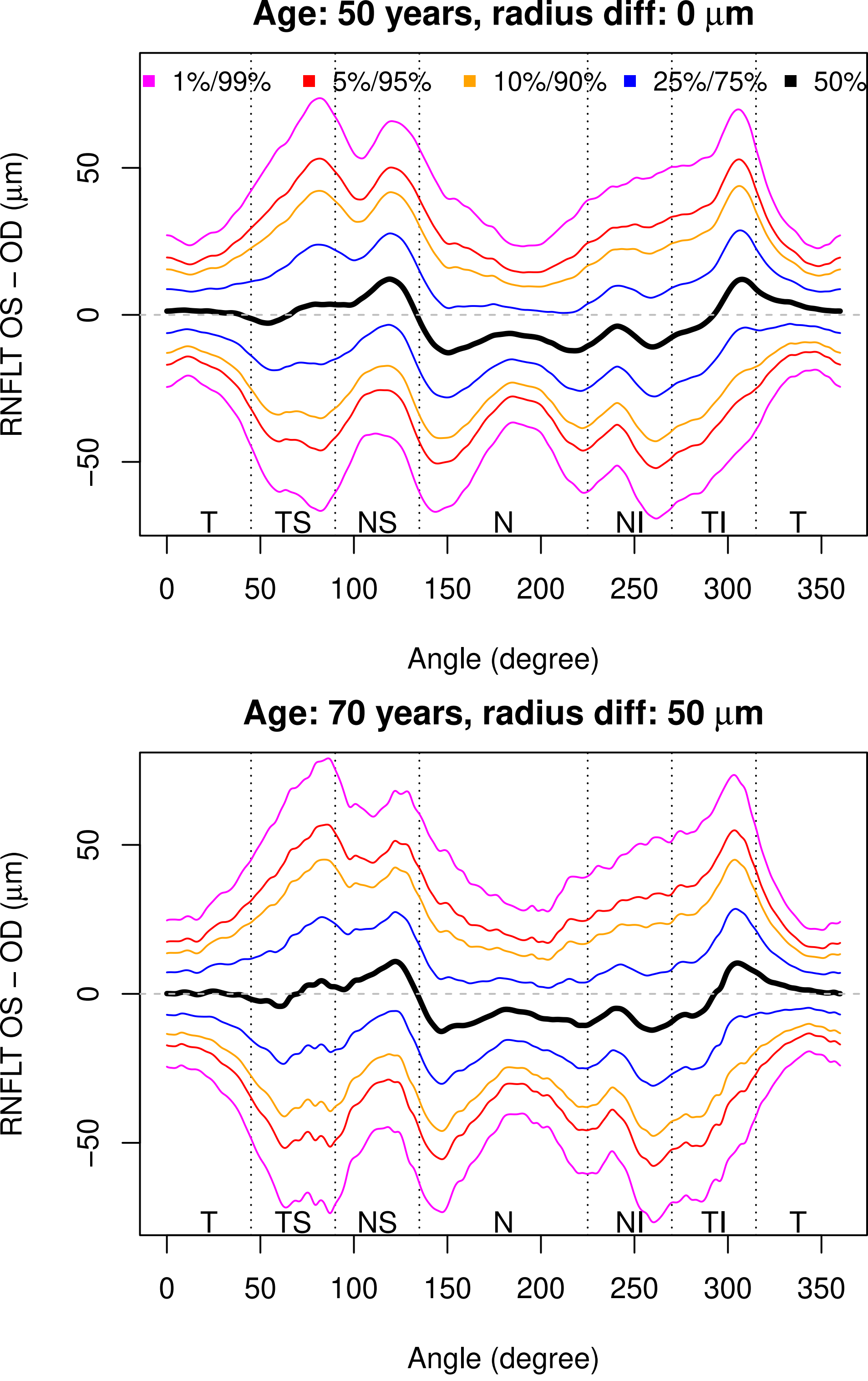

Supplementary Figure S8: Illustrative examples of personalized norms for 50-year olds with equal scanning radii in both eyes (top panel) and for 70-year olds with a radius difference of 50 μm. Our supplemental software allows the generation of such normative plots for any ages and scanning radius differences, based on the numerical values which we provide in a public repository.
