# Supplementary material for: Norms of Interocular Circumpapillary Retinal Nerve Fiber Layer Thickness Differences at 768 Retinal Locations
Source: Transl Vis Sci Technol. 2020 Aug 12;9(9):23. doi: 10.1167/tvst.9.9.23 (PMC7442876; doi:10.1167/tvst.9.9.23)
Supplement: Supplement 8 [file tvst-9-9-23_s008.pdf]

Table S5 Demographics: 4430 (98.8%) of the subjects were European.

Parameters	n
Age categories:	
19-39 y	312
40-79 y	4171
Sex:	
Female	2,466
Male	2,017
Ethnicity:	
European	4430
Arabic/Middle Eastern	11
African	4
Central Asian	8
East Asian	3
Latin American	4
Mixed or other descent	23
Total	4483
